# 2295: A novel in vivo zebrafish model of hematopoietic stem cell-driven regeneration of blood

**DOI:** 10.1017/cts.2017.35

**Published:** 2018-05-10

**Authors:** Samima Sultana Habbsa, Mia McKinstry, Sara Payne, Christian Mosimann, Teresa Bowman

## Abstract

OBJECTIVES/SPECIFIC AIMS: Hematopoietic stem and progenitor cells (HSPCs) function to maintain steady state production of new blood cells and to rapidly respond to blood cell loss. Little is known regarding how HSPCs develop the ability to sense and respond to blood cell loss during embryogenesis. Gaining insight into the robust ability of HSPCs to regenerate blood during early development may allow us to develop therapies to rejuvenate this capacity at any stage. METHODS/STUDY POPULATION: We generated a new hematopoietic-specific and inducible cell ablation zebrafish model to uncover the origins of regenerative capacity in HSPCs during development. These transgenic zebrafish express a cyan fluorescent protein (CFP)-nitroreductase (NTR) fusion construct under the control of the draculin (drl) promoter (drl:CFP-NTR), which restricts NTR expression to blood cells. Co-expression analyses of drl:CFP-NTR with known markers of other blood types including HSPCs (runx1+23:mCherry), erythroid cells (gata1:dsRed), and lymphoid cells (rag2:RFP), revealed drl:CFP-NTR was restricted to HSPCs and erythrocytes. To delineate the regeneration potential of embryonic HSPCs, we exposed drl:CFP-NTR transgenic zebrafish embryos to Metronidazole (MTZ), which results in selective ablation of only NTR-expressing blood cells. Embryos were treated from 2 to 3 days postfertilization and recovery of drl+ and gata1+ cells was evaluated over a 7-day recovery period. RESULTS/ANTICIPATED RESULTS: Following MTZ exposure, the nadir of drl+ cell ablation occurs at 2 days post MTZ (dpM) treatment. During the renewal phase of blood regeneration, we first observe recovery of drl+ cells by about 4 dpM, while more mature blood cells like gata1+ erythrocytes show a delayed recovery at about 6 dpM. Our results suggest that HSPCs can respond to injury as early as 2 days of life and that the HSPC-driven regeneration of embryonic blood cells occurs in a hierarchical fashion, similar to regeneration of the adult blood system. DISCUSSION/SIGNIFICANCE OF IMPACT: We have established a quantitative method for in vivo real-time monitoring of embryonic and larval blood regeneration. A significant advantage of our system is that we can use these insights to guide an in-vivo drug screen for factors that accelerate HSPC-driven blood regeneration in a complex biological environment.

